# Studies on the Cooperative Influence of Cr and Mo on the Pitting Corrosion Resistance of Super Austenitic Stainless Steels

**DOI:** 10.3390/ma16237397

**Published:** 2023-11-28

**Authors:** Bingbing Li, Yuping Lang, Haitao Chen, Huapeng Qu, Hanqiu Feng, Xu Sun, Zhiling Tian

**Affiliations:** 1 Central Iron and Steel Research Institute Company Limited, Beijing 100081, China; libingbing66@foxmail.com (B.L.);; 2 Material Digital R&D Center, China Iron & Steel Research Institute Group, Beijing 100081, China

**Keywords:** Cr, Mo, passive film, first-principles, pitting corrosion resistance, super austenitic stainless steel

## Abstract

The effects of varying Cr and Mo concentrations on the pitting corrosion resistance of super austenitic stainless steels in Cl^−^ solutions were investigated using a combination of immersion experiments, electrochemical measurements, X-ray photoelectron spectroscopy, and first-principles computational simulations. The surface characteristics, impedance, and defect concentration of the passive film were changed, and this eventually resulted in a decrease in the number of pitting pits. Due to a decrease in active sites within the passive film, a delayed beginning of pitting, and the combined effect of MoO_4_^2−^ inhibitors, it was discovered that an increasing Mo concentration slows the rate of pitting extension, resulting in reduced maximum pitting area and depth. Additionally, Mo increased the adsorption energy of nearby atoms, whereas Cr raised the adsorption energy of itself. Interestingly, compared with individual doping, co-doping of Cr and Mo increased work function and adsorption energy, indicating a synergistic impact in enhancing resistance to Cl^−^ corrosion.

## 1. Introduction

Super austenitic stainless steels are used widely in pulp and paper, flue gas desulphurization, petrochemicals, and other industries because of their high Cr, Mo, and N content and ability to successfully resist crevice corrosion, pitting corrosion, and uniform corrosion [1]. Passive coatings on the surfaces of super austenitic stainless steels, which are mostly made up of the oxides and hydroxides of elements like Cr and Fe, especially Cr_2_O_3_, determine how resistant they are to corrosion. A passive film can reduce or separate stainless steel from a corrosive environment medium, resulting in a corrosion resistance function [2]. The pitting resistance equivalent number (PREN) value has several expressions, the most popular of which is PREN = 1 × wt.% Cr + 3.3 × wt.% Mo + 16 × wt.% N [3], and it can be used to assess the local corrosion resistance of super austenitic stainless steels. According to its equation, the amount of Cr, Mo, and N in super austenitic stainless steels affects how resistant they are to local corrosion.

Latha et al.’s research [4] on the function of Cr in passivation films revealed that Cr is enriched on the alloy surface as CrOOH, which enhances a passive film’s ability to resist corrosion. While Clayton et al. [5] noted that the majority of the Cr in a passive film is present as XCr_2_O_3_-YCrO_3_ in the glassy state, Brooks et al. [6] came to the opposite conclusion, concluding that the presence of Cr in the form of CrO_4_^2−^ ions functions as an inhibitor to enhance the corrosion resistance of stainless steel. Yaniv et al. [7] noted that the favorable effect of Mo on a passive film is to enhance the bonding quality at the metal–oxide interface, thus generating a barrier layer. According to some experts, Mo produced MoO_4_^2−^ as it dissolved on the metal surface, thereby preventing Cl^−^ from adhering to it [8,9]. In contrast, several specialists concluded that Mo, in the hydrated forms of MoO_2_, MoO_4_^2−^, and MoO(OH)_2_, is present in passive films and contributes to their homogeneity [10]. According to Sugimoto et al. [8], the addition of Mo enhances a passive film’s protective qualities since hexavalent molybdenum oxide has a high degree of stability in acidic chloride solutions. According to Hashimoto et al. [11], the principal function of Mo is to lower the pace at which the active zone dissolves by producing molybdenum oxide, or MoO_4_^2−^.

Studies by Ilevbare and Burtstein [12] showed that the presence of Mo decreases the likelihood of forming stable pitting pits and reduces the size and quantity of nucleation and sub-stable pitting pits in stainless steels. According to several studies, molybdenum (Mo) enhances re-passivity by generating insoluble MoCl_2_ complexes [13] or MoO_4_^2−^ [14] at active sites. The adsorption of MoO_4_^2−^ in pitting pits may hinder the early growth phase of pitting pits, according to Sugimoto and Sawada [15]. According to research by Ha et al. [16], the addition of Mo can enhance a passive film’s protection and lower the rate of pitting expansion, which increases pitting resistance.

On passive films, Cr and Mo exhibit a variety of advantageous effects, as well as synergistic effects. Lu et al. [17] proposed that Mo alters the polarity of passive films by forming MoO_4_^2−^, resulting in a passive film consisting of a cation-selective exterior layer and an anion-selective interior layer, which enhance the deprotonation of Cr(OH)_3_, hence preventing Cl^−^ from entering a passive film and making it more stable [18] and promoting the migration of O^2−^, which facilitates the formation of Cr_2_O_3_ or CrO_3_ [19]. Habazaki et al. [20] concluded that Mo raises the Cr content in passive films or encourages the production of Cr_2_O_3_ to improve corrosion resistance. According to Jung et al.’s observation [21], the simultaneous co-dissolution of Fe and Mo during the passivation process is encouraged by the presence of Mo, which also encourages an enrichment in Cr in a passive film. Montemor et al. [9] considered that the chromium oxide layer in passive film defects generated by Fe^2+^ was counteracted by Mo^4+^ and Mo^6+^.

In the context of marine oil and gas extraction, where chloride ions are abundant, super austenitic stainless steel is particularly susceptible to pitting corrosion. To economize on materials and extend the operational life of equipment, it is imperative to enhance the corrosion resistance of super austenitic stainless steel by optimizing the balance of corrosion-resistant elements. The PREN primarily depends on the levels of Cr, Mo, and N. Nevertheless, a simplistic strategy of independently increasing the content of Cr, Mo, and N content is not advisable. This is because higher chromium content can trigger the formation of ferrites, excessive molybdenum may heighten sensitivity to thermal cracking, and nitrogen’s solubility at atmospheric pressure is limited. While extensive research has explored the effects of individual elements and a few studies have investigated synergistic interactions, these investigations have mainly focused on adjusting the elemental composition without establishing fixed PREN values.

This study examines the cooperative influence of Cr and Mo on pitting corrosion propagation in super austenitic stainless steels by adjusting the Cr and Mo content to achieve identical PREN values. The effect of Cr and Mo on pitting corrosion is examined using immersion tests, and the characteristics of the passive layer that forms on the steel surface are examined using electrochemical methods. Additionally, XPS and TEM studies are used to determine the composition and thickness of the passive film, and DFT simulations are used to show how Cr and Mo work in concert. These analyses revealed the mechanisms underlying the Cr-Mo synergy in super austenitic stainless steels’ resistance to Cl^−^ ions.

## 2. Experimental Methods

### 2.1. Material Preparation

**Figure 1:**
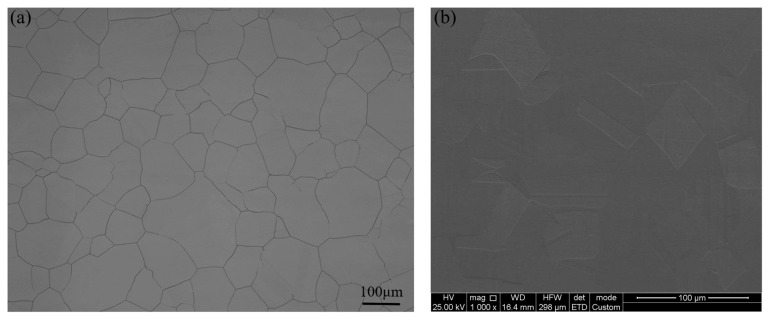
Metallographic structure and microstructure of the test steel: (**a**) metallographic organization and (**b**) microstructure.

Design plan proportions were followed when mixing pure metal in a vacuum induction furnace to create the pilot sample. The vacuum was then released, iron chromium nitride was added, and deoxidation with Ni-Mg was carried out afterward. The alloy was then cast, forged, and rolled into sheets with a thickness of 5 mm. There was an unavoidable loss of metallic components during the forging and hot rolling at 1150 °C. As shown in Table 1, the final component of the samples was identified with quantitative analysis utilizing inductively coupled plasma atomic emission spectrometry. The samples, which were provided in the hot-rolled form, underwent a heat treatment at 1200 °C for two hours to achieve a pure austenitic structure. This structure was then retained with swift liquid cooling [22,23]. The metallographic structure and microstructure of the test steel are shown in Figure 1.

### 2.2. Research Methods for Pitting Pits and Re-Passivation

#### 2.2.1. Immersion Corrosion Method

The samples were quickly desiccated in a drying container after being rinsed with an ultrasonic cleaner. Their surface area was precisely measured, and then they were weighed after 24 h of standing. Two parallel samples from each trio were immersed in a 6% FeCl_3_ + 0.05 mol/L HCl solution for 12 h at 75 °C. The samples were then dried, shaken with alcohol, and rinsed with distilled water. A Bruker ContourX-500 (Bruker, Billerica, MA, USA) was used to measure the 3D morphology of the pitting pits, and Vision64 software (5.60 Update 1) was used to statistically analyze the data. The corrosion rate was assessed against the loss-of-weight corrosion rate (g/(m^2^·h)) of GB/T 17897-2016. To ensure the accuracy of the experiments, a total of 30 samples were taken from each group.

#### 2.2.2. Electrochemical Method

A saturated calomel electrode (SCE), a platinum sheet, and a 1 cm^2^ polished sample surface were used as the working, reference, and counter electrodes in standard three-electrode electrochemical studies using a Gamry 1000 electrochemical system (Gamry Instruments, Warminster, PA, USA). The sample was pre-polarized at −0.8 V (vs. SCE) cathode for 10 min and passing nitrogen gas for 20 min, and 0.5 L/min of nitrogen gas was continuously passed during the experiment to eliminate dissolved oxygen from the solution and surface oxide film.

To evaluate susceptibility to pitting corrosion and the ability to re-passivate the samples, cyclic polarization tests were conducted at a temperature of 75 °C in a solution comprising 6% FeCl_3_ and 0.05 mol/L HCl with a scan rate of 20 mV/min [24]. As soon as the current reached 1 mA, a reverse sweep was initiated, halting at the point where it intersected with the forward scanning curve.

### 2.3. Methods for the Production and Electrochemical Measurement of Passive Films

Potentiodynamic was carried out in a 3.5% NaCl solution at 50 °C with a scan rate of 20 mV/min, sweeping from −0.6 V to 1.0 V (vs. SCE) [24]. Additionally, potentiostatic measurements were taken for an hour at the same temperature with a constant potential of 0.3 V (vs. SCE).

Electrochemical impedance spectroscopy (EIS) and Mott–Schottky (M-S) techniques were used to assess the impedance and semiconductor properties of the passivated layer. The data were fitted and analyzed using Zsim software (3.30 d), and the frequency range of the EIS was 10^5^ Hz to 10^−2^ Hz with a positive frequency orientation and an amplitude of 5 mV. M-S had a frequency of 2 kHz, a scan speed of 50 mV/s, a negative potential orientation, and a voltage scope of 1 to −0.5 V.

### 2.4. Methods for Visualizing the Morphology of the Passive Film

TEM specimens were prepared using a GATAN PIPS II 695 precision ion thinning device (Gatan, Walnut Creek, CA, USA). To be more definite and lessen the inconsistent thickness of the passivation film formed by other causes, the passive film was generated in a very short period due to the comparatively dense composition of Cr, Mo, and N in the experimental steel [25]. An examination of the raw passive film was conducted immediately after part of the sample preparation was finished, and another portion of the sample preparation was finished and immersed in a 3.5% NaCl solution at 50 °C for 24 h. A transmission electron microscope, model FEI Tacnai F20 (FEI, Hillsboro, OR, USA), was used for the TEM test.

### 2.5. A Research Approach to Passive Film Components

After performing potentiostatic measurements using X-ray Photoelectron Spectroscopy (XPS) equipment (Shimadzu, Kyoto, Japan) with a Kratos Axis UltraDLD, the compositional structure of the passive film was calculated using Factasge and the E-pH diagram of the Fe-Cr-Mo system in aqueous solution at 50 °C. For XPS, the 1s peak of C (284.8 eV), which is a single-color Al K ray, serves as a correction for other photoelectron peak binding energies. The voltage, power, take-off angle, analysis area, and sputtering rate in comparison to SiO_2_ were 1486.6 eV, 150 W, 45°, 700 µm × 300 µm, and 3 nm/min. For subpeak fitting and background subtraction correction, XPS peak version 4.1 and Shirley-type were used.

### 2.6. Numerical Simulation Research methodology

To maintain the ionic nature of Cl^−^, NaCl molecules were used to represent the surface adsorption energy of passive films. By analyzing the adsorption behavior of NaCl, the construction of Cr and Mo-doped Fe_2_O_3_ surfaces and the mechanism of Cl^−^ corrosion resistance of passive films were examined. Using the Vienna Simulation Package (VASP), spin-polarized Density Functional Theory (DFT) calculations were made [26]. The PBE variant of the generalized gradient approximation (GGA) was used to represent exchange correlations among electrons [27]. Using Hubbard U corrections of 4 eV, 5.5 eV, and 5.5 eV for Fe, Cr, and Mo, respectively, the GGA + U approach was used to account for the in situ Coulomb interaction of d-orbital electrons [28,29]. A plane wave cut-off energy of 450 eV and an energy convergence threshold of 10^−5^ eV were used in all calculations. The 4 × 4 × 1 and 4 × 4 × 3 Brillouin zones were used, respectively, for the surface and bulk models [30]. Up until the stress on all atoms was less than 0.02 eV/A, the top 8 atomic layers loosened while the bottom 10 atomic layers were immobilized. With a vacuum gap of 15 Å from the surface of (0001), the antiferromagnetic order between Fe atomic layers in Fe_2_O_3_ was + + − −. The Bader charge was measured to obtain the amount of atomic charge transfer [31]. Due to the asymmetric top surface and bottom surface electronegativity difference after doping, which will produce additional dipole torque in the vicinity of two surfaces and cause an inaccurate measurement of vacuum electrostatic potential, dipole correction was applied when calculating the work function [32].

## 3. Results and Discussion

### 3.1. Cr and Mo Doping Influence on Pitting Corrosion Holes

**Figure 2:**
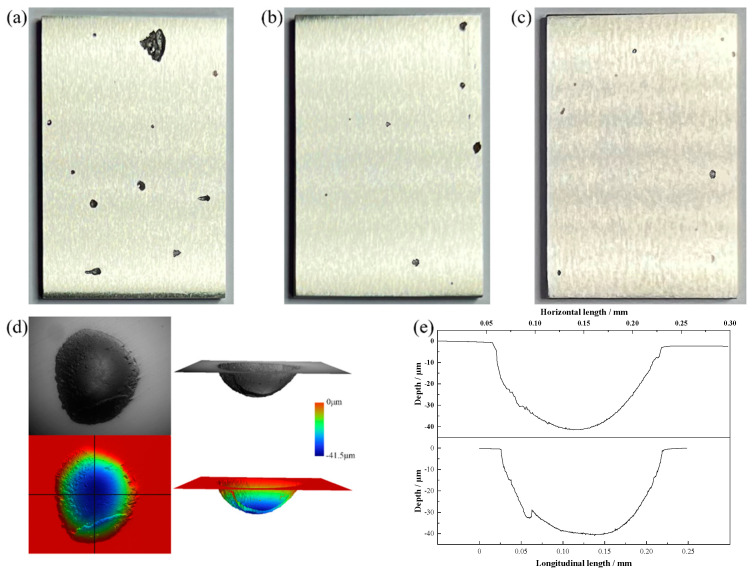

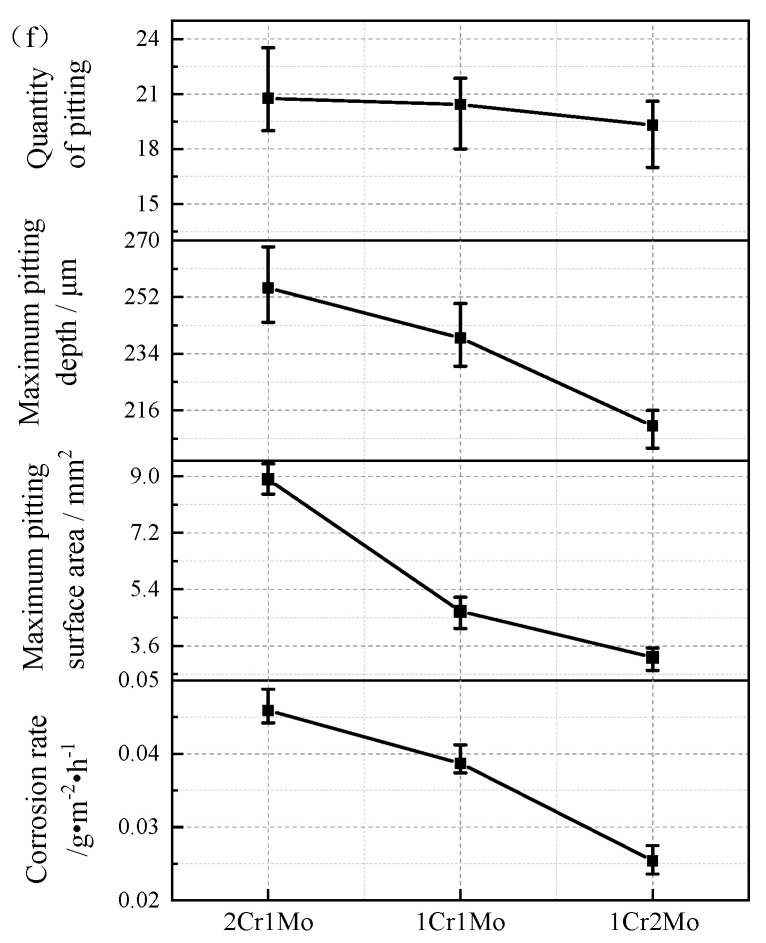
Surface of the specimen after immersion corrosion, morphology and parameters of small pitting corrosion pits, parameters of specimen corrosion. (**a**) Cr2Mo1, (**b**) Cr1Mo1, (**c**) Cr1Mo2, (**d**) morphology of small pitting corrosion pits, (**e**) parameters of small pitting corrosion pits, and (**f**) parameters of specimen corrosion.

Figure 2a–c shows the small pitting corrosion pits that formed on the specimen surface during immersion exhibited circular shapes, while the larger pitting pits appeared irregular. The 3-D shape of a typical small pitting pit is illustrated in Figure 2d,e. No self-sealing water droplet corrosion pits were discovered; instead, all the pits were open and circular, with their radii being larger than the depth orientation. This can be attributed to the accumulation of reaction by-products inside the pitting pits, impeding their further expansion. Moreover, the inclusion of N in the tested steels inhibited a strong autocatalytic effect within the pitting pits. The relatively short duration of corrosion could also be a contributing factor to this occurrence.

The corrosion parameters associated with the immersion of the three samples in a 6 % FeCl_3_ + 0.05 mol/L HCl solution at 75 °C for 12 h are shown in Figure 2f. As can be seen from Figure 2f, the corrosion rate, maximum pitting area, maximum pitting depth, and the number of pitting pits all decreased as Mo concentration increased and Cr concentration decreased, indicating an improvement in the test steels’ corrosion resistance.

The decrease in the count of pitting corrosion pits, aligning with higher Mo concentration and lower Cr concentration, can be ascribed to modifications in the composition and structure of the passive film formed on the specimen’s surface. The variation in alloy composition prompts alterations in the passive film, leading to a reduction in surface imperfections, a decrease in active pitting corrosion sites, and a postponement in the onset of pitting corrosion under identical environmental circumstances.

Moreover, augmenting the Mo concentration while diminishing the Cr concentration results in a decrease in both the maximum pitting area and the maximum pitting depth caused by pitting corrosion. This decline can be attributed to a reduced rate of propagation of pitting pits, either accelerating re-passivation or postponing the commencement of pitting corrosion. To grasp the fundamental causes of these changes, we initiated a cyclic polarization test.

**Figure 3:**
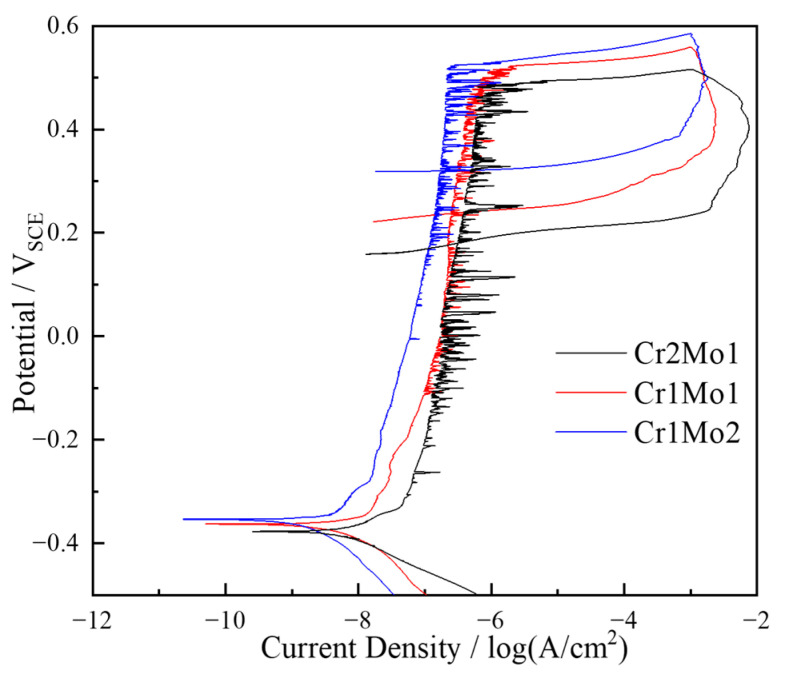
Cyclic polarization curves in a 6% FeCl_3_ + 0.05 mol/L solution at 75 °C.

Figure 3 depicts cyclic polarization curves for the three test steels in a 6% FeCl_3_ + 0.05 mol/L solution at 75 °C. In Figure 3, it is clear that there is no active-to-passive transition present, but rather, a temporary passive state [33]. As the Mo concentration rises from 4.02 wt.% to 6.02 wt.% and the Cr concentration falls from 27.96 wt.% to 21.45 wt.%, the critical current density decreases, the self-corrosion potential gradually rises, and the pitting corrosion potential slightly increased. These findings suggest that a higher Mo concentration and a lower Cr concentration led to a reduced likelihood of pitting corrosion pit formation. This correlation is associated with the composition and structure of the passive film, as explained in the subsequent section.

The passive areas on the surfaces of all three specimens display distinct current fluctuations, implying the presence of numerous sub-stable pitting corrosion. As the Mo concentration increases and the Cr concentration decreases, these current fluctuations are initially noticed at more elevated potentials, but they occur less frequently and exhibit reduced transient current amplitudes. This implies that these alterations hinder the development of sub-stable pitting corrosion pits. This observation is in line with the declining trend we observed in the count of pitting corrosion pits during the immersion corrosion test.

After the current reached 1 mA, the potential was reversed. Then, the current density peaked and then gradually decreased, even as the applied potential decreased. This pattern formed a hysteresis loop, indicating a delay in the re-passivation process for pitting corrosion [34]. We observed a distinct increase in the potential required for re-passivation, indicating that a higher Mo concentration and a lower Cr concentration increased the susceptibility of pitting pits to undergo re-passivation. This implies a notable slowdown in the propagation rate of the pitting pits. Moreover, the difference between the potential for pitting and the potential for re-passivation decreased gradually, indicating an enhancement in re-passivation kinetics [16]. The area of the hysteresis loop is a direct measure of the pit propagation kinetics [35].

The area of the hysteresis loop rapidly shrinks with decreasing Cr content and increasing Mo content, suggesting that the spread of the pitting corrosion pits is hindered. This was attributed to the production of the stable micro-soluble compound MoO_4_^2−^ by Mo, which precipitates in the dissolved areas on the inner surface of the pitting pits and may have reduced the relative anodic area and hence inhibited the expansion of pitting pits [36]. This is consistent with the decreasing trend in the maximum pitting pit area, depth, and weight loss rate in the immersion corrosion test.

### 3.2. The Influence on the Polarization of Cr and Mo Concentrations

The composition and shape of the passive film may alter with rising Mo concentration and falling Cr content, which would lessen the quantity of pitting corrosion pits. Additionally, lowering the Cr content and raising the Mo concentration may delay the commencement of pitting corrosion, which would reduce the maximum pitting depth and area. Therefore, a comprehensive investigation of the passive film was performed.

**Figure 4:**
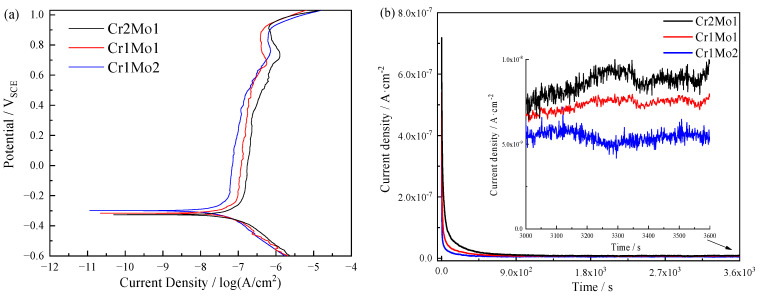
Polarization curves in 3.5 % NaCl liquor at 50 °C: (**a**) potentiodynamic polarization curves and (**b**) potentiostatic polarization curves.

The potentiodynamic polarization of the three specimens in a 3.5% NaCl solution at 50 °C is shown in Figure 4a. When the polarization of specimens is observed in Figure 4a, it is clear that there is no active-to-passive transition present, but rather, a temporary passive state [33] with all specimens having a sizable passive zone up until transpassive. The passive current density and critical current density decrease when the Mo concentration rises from 4.02 wt.% to 6.02 wt.% and the Cr concentration falls from 27.96 wt.% to 21.45 wt.%, whereas the self-corrosion potential exhibits steadily rising patterns. This shows that the Cr and Mo concentrations affect the test steel’s corrosion resistance synergistically and that the PREN’s simple summing does not adequately account for their impact.

Passive films were created by applying 0.3 V (vs. SCE) for 1 h at 50 °C in a 3.5% NaCl solution to look into the cause of it. Figure 4b shows the transient current density of the three samples and emphasizes the dramatic drop in current density brought on by the removal of the surface passive film during the pre-test phase. The slower melting speed of the specimen surface compared with the rate of passive film formation resulted in the passive film continuing to accumulate on the surface at an applied potential of 0.3 V (vs. SCE) [37]. The current density gradually stabilized with time. The three specimens’ surfaces were completely passivated and remained remarkably stable, as shown in the magnified portion of Figure 4b. The stable current density of the specimens decreases as the Mo concentration rises and the Cr concentration falls, demonstrating the enhanced protective role of the passive film [38], probably as a result of changes in the passive film’s constituents brought on by the different Cr and Mo concentrations.

### 3.3. Impact of Cr and Mo Concentration on the Impedance of Passive Film

**Figure 5:**
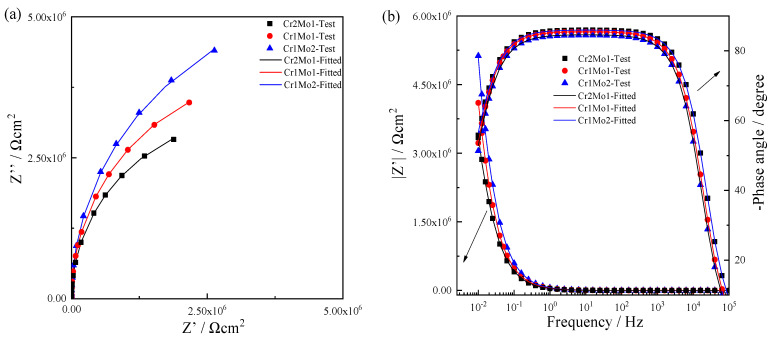
EIS diagram of specimens in a 3.5% NaCl medium at 50 °C: (**a**) Nyquist and (**b**) Bode.

EIS tests were performed on three specimens in 3.5% NaCl solution at 50 °C after applying a static potential of 0.3 V (vs. SCE) for 1 h. The results are shown in Figure 5. Using a Kramers–Kronig (K-K) transformation to swap the real axis for the imaginary axis and the imaginary axis for the real axis, the correctness of the EIS data was examined, and all transformation deviations from the experiment datum were less than 10^−3^ [39].

The three specimens’ Nyquist plots in Figure 5a all show arcs, indicating that they have similar impedance characteristics in a 3.5% NaCl solution at 50 °C. The phase angle in Figure 5b is constantly toward 90° but somewhat below 90°, showing that the three specimens’ capacitive characteristics deviate from the ideal capacitive characteristics, which can be represented by a constant phase element (CPE) [40]. Additionally, elements like surface quality and porosity have an impact on capacitive features [5,6].

The impedance of the CPE can be defined by the fitting parameter Q, the angular frequency ω (rad/s), the imaginary number j^2^ = −1, and the CPE index n:

Z_CPE_ = 1/Q(jω)^n^ (1)

When n = 1, the CPE is expressed as the ideal capacitance; when n = 0, the CPE is expressed as the resistance; and when n = 0.5, the CPE is expressed as the Warburg impedance. n varies closely with the smoothness of the passive film [41].

**Figure 6:**
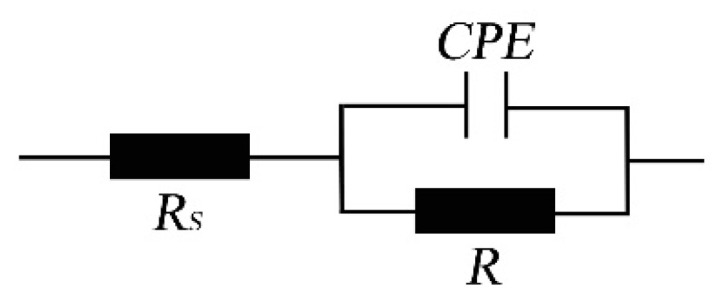
Circuit construction diagram.

Figure 6 depicts the circuit topology of the equivalent circuit model fit that was performed on the measurement data, with R_S_ standing for solution resistance and R standing for passive film resistance.

The equivalence circuit simulation data are shown in Table 2. *n* and *R* gradually get better when the Mo level rises and the Cr level falls. When the Mo concentration is 6.02 wt.% and the Cr concentration is 21.45 wt.%, the passive film is the tightest it can be. This finding explains the potentiostatic test and demonstrates how variations in Cr and Mo concentration impact the passive film’s tightness. Equation (2) can be used to calculate the capacitance *C* based on the parameters *Q*, *n*, and *R* obtained from the circuit fit [42]. The thickness of the oxide layer of the steady-state passivate film, d_OX_, can then be determined using Equation (3) and *C* [43].

(2) $$C=\sqrt[{n}]{\frac{Q}{R^{n-1}}}$$

(3) $$d_{ox}=\frac{\varepsilon \varepsilon_{0}A}{C}$$

where the vacuum dielectric constant ε_0_ is 8.854 × 10^−14^ F/cm, the dielectric constant ε is 15.6 [44,45,46], and the effective area *A* is measured in m^2^. It is difficult to estimate the thickness of the passive film precisely because of how thin it is and the fact that its outer layer is made up of porous hydroxides or hydrates of iron and chromium [47]. Nevertheless, for certain specimens, *C* can reveal the dox tendency, with rising Mo concentrations and falling Cr concentrations representing an increase in dox as C declines.

### 3.4. Influence of Cr and Mo Concentration on the Semiconductor Behavior of Passive Films

Passive films on the surface of stainless steel exhibit the characteristics of highly doped semiconductors, of which those with oxygen vacancies or cation interstitials are known as n-type semiconductors, and those with cationic vacancies are known as p-type semiconductors [44]. The capacitance of a semiconductor can be represented by the expression:

(4) $$\frac{1}{C}=\frac{1}{C_{SC}}+\frac{1}{C_{H}}$$

where C_sc_ stands for space charge capacitance, while C_H_ stands for Helmholtz capacitance. C_sc_ is extremely small by comparison with C_H_ when a disturbance of high-frequency potential is imposed, so the measured interface capacitance may neglect C_H_ and treat it as C_sc_. Thus, the relationship between space charge capacitance and potential for p-type and n-type semiconductors can be deduced as follows:

(5) $$\frac{1}{C_{cs}^{2}}=\frac{-2}{\varepsilon \varepsilon_{0}eN_{A}}\left(E-E_{fb}-\frac{kT}{e}\right)$$

(6) $$\frac{1}{C_{cs}^{2}}=\frac{2}{\varepsilon \varepsilon_{0}eN_{D}}(E-E_{fb}-\frac{kT}{e})$$

where the dielectric constant ε is 15.6, the vacuum dielectric constant ε_0_ is 8.854 × 10^−14^ F/cm and the electron charge is *e* is 1.602 × 10^−19^ C [48]. The n-type semiconductors exhibit electron donor concentrations of *N_D_* and the p-type semiconductors exhibit vacancy acceptor concentrations of *N_A_*, both in cm^−3^ units, which can be found from the slope of the C^−2^-E. The absolute temperature is T, the Boltzmann constant is k of 1.38 × 10^−23^ J/K, the applied voltage is E, and the flat-band potential E_FB_ can be derived by extrapolating to C^−2^ = 0.

**Figure 7:**
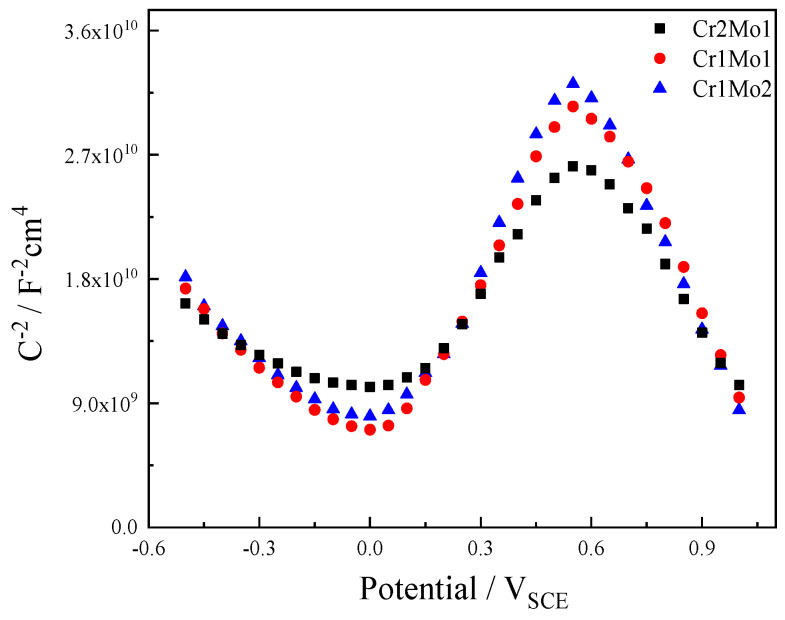
M-S pictures in 3.5% NaCl liquor at 50°C.

Figure 7 shows the M-S pictures of the three specimens after applying 0.3 V (vs. SCE) for 1 h at a fixed potential in a 3.5% NaCl liquid at 50 °C. The figures show that each specimen has identical properties, which suggests that passive films have comparable electrical attributes. The C^−2^-E slope is positive for potentials between 0.55 V (vs. SCE) and 0.0 V (vs. SCE), indicating n-type semiconductor identities. The slope of C^−2^-E is negative in the negative potential region, indicating p-type semiconductor identities. This exemplifies the passive film’s dual nature, which is generally thought to consist of an inner layer of chromium oxide and an outside layer of chromium oxide [49].

The N_A_, N_D_, and E_FB_ values calculated from the slope of the M-S diagram are listed in Table 3. As the Mo content increases and the Cr concentration drops, N_A_ and N_D_ drop. The defect density of the passive film decreases, the conductivity of the space charge decreases, and interfacial reactions become more difficult to initiate as the Mo content rises and the Cr concentration falls [50]. A rise in E_FB_-n simultaneously reduces the transfer of electrons to the solution and the amount of positive charge on the surface of the passive film, which attenuates the electrostatic attraction from the surface to the Cl^−^ [30]. This is because the n-type semiconductor is outside of the passive film and in contact with the solution.

### 3.5. Influence of Cr and Mo by Components and Thickness of Passive Films

**Figure 8:**
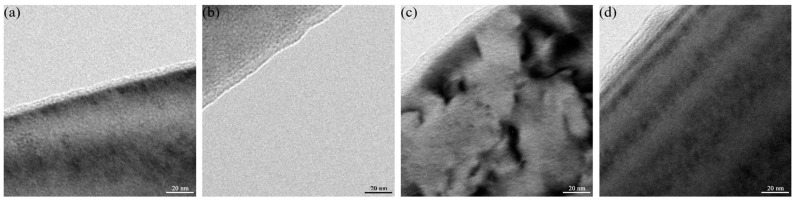
TEM pictures of passive films after 24 h of immersion in 3.5% NaCl at 50 °C: (**a**) unsoaked specimen of Cr1Mo2, (**b**) Cr2Mo1, (**c**) Cr1Mo1, and (**d**) Cr1Mo2.

Figure 8a shows the Cr1Mo2 sample’s passive film before immersion treatment, while Figure 8b–d shows the passive films following immersion in a 3.5% NaCl solution at 50 °C for 24 h. Table 4 shows the thicknesses of the passive films measured at the five positions and their average values. It is clear from Figure 8 and Table 4 that the passive film on the soaked samples is thicker than that on the unsoaked samples, and that the passive films of Cr1Mo2 are thicker, which may explain why the Cr1Mo2 samples had less potentiostatic current, more resistance, and smaller N_A_ and N_D_.

**Figure 9:**
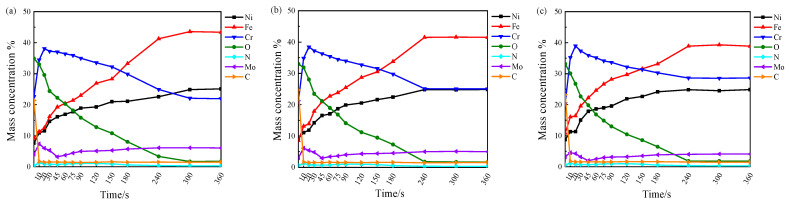
Depth distribution of various specimen elemental consistencies in a 3.5% NaCl liquid at 50 °C: (**a**) Cr2Mo1, (**b**) Cr1Mo1, and (**c**) Cr1Mo2.

For a 3.5% NaCl liquid at 50 °C, Figure 9 shows the depth distribution of the elemental content of the passive film for the three specimens. It is observed that C, a complex organic compound, is located on the surface of the passive film and is prevalent in the contaminated layer [51], with the quantity of C rapidly decreasing as sputtering progresses. The ratio of Cr to O in the three specimens grows sequentially according to Cr2Mo1, Cr1Mo1, and Cr1Mo2 at the same sputtering time, suggesting that the passive film thickness increases sequentially, in agreement with the prior findings for the passive film thickness in Figure 8 and Table 4. As the sputtering duration continues, the Fe, Ni, and Cr concentrations of the three samples gradually converge toward the matrix concentration.

**Figure 10:**
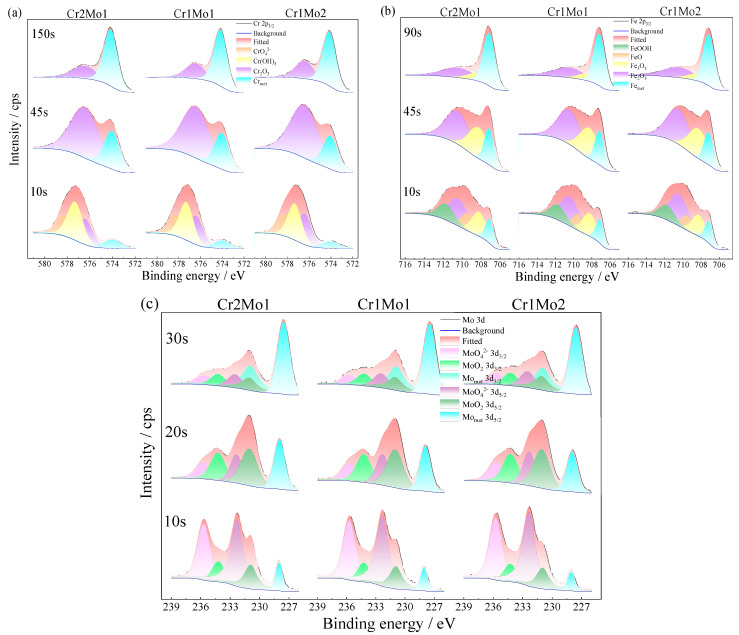
Cr2p3/2, Fe2p3/2, and Mo3d time varying photoelectron spectra: (**a**) Cr2p_3/2_, (**b**) Fe2p_3/2_, and (**c**) Mo3d.

**Figure 11:**
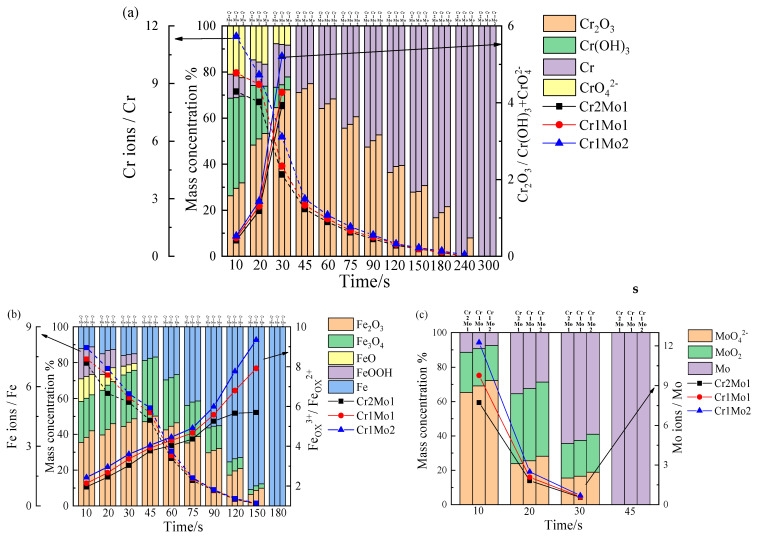
Concentration and ratio (**a**) Cr, Cr ions/Cr and Cr_2_O_3_/Cr(OH)_3_ + CrO_4_^2−^; (**b**) Fe, Fe ions/Fe and Fe_OX_^3+^/Fe_OX_^2+^; and (**c**) Mo, Mo ions/Mo.

Table 5 provides the photoelectron spectral binding energies for Cr2p_3/2_, Fe2p_3/2_, and Mo3d [5,7,52,53]. Figure 10 and Figure 11 are fitted diagrams of the photoelectron spectra of Cr2p_3/2_, Fe2p_3/2_, and Mo3d.

**Figure 12:**
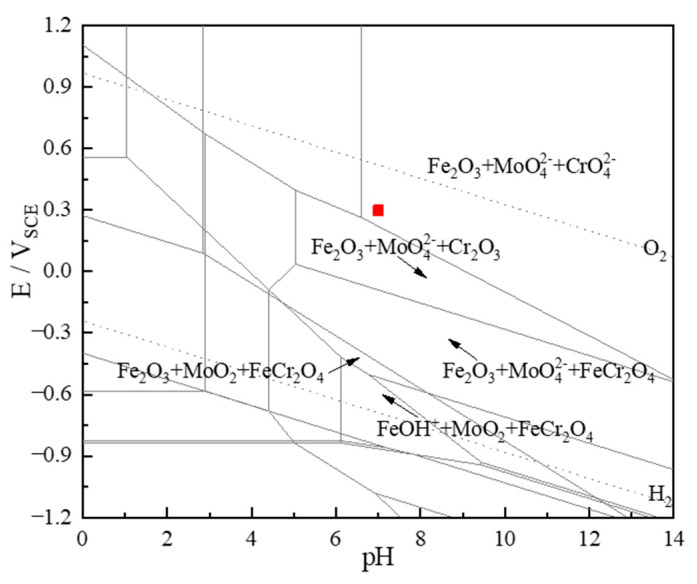
Pourbaix graph of the alloy of the Fe-Cr-Mo system in aqueous solution at 50 °C. (The red part is the experimental condition of this manuscript).

Figure 10 and Figure 11 reveal that the predominant components of Cr2_p3/2_ include CrO_4_^2−^, Cr(OH)_3_, Cr_2_O_3_, and Cr_met_, the predominant components of Fe2_p3/2_ include Fe_2_O_3_, FeO, Fe_3_O_4_, Fe_met_, FeOOH, and the predominant components of Mo3d are MoO_4_^2−^, MoO_2_, Mo_met_. The Pourbaix graph (Figure 12) shows that Fe, Cr, and Mo form stabilized complexes CrO_4_^2−^, Fe_2_O_3_, and MoO_4_^2−^ under neutral circumstances at 0.3 V (vs. SCE). The fact that XPS finds more complexes than Pourbaix predicts suggests that passive film development is a kinetic equilibrium process involving complex production and resolution. The Pourbaix diagram is based on thermodynamic values for known bulk compounds, which is not surprising. Although thermodynamic forecasts cannot completely anticipate the complexes in the passive film, the general trend is in line with thermodynamic projections. In the lower right of 0 V (vs. SCE), there is a large stable region of FeCr_2_O_4_, which is probably composed of FeO·Cr_2_O_3_ with a spinel structure that can act as a barrier to pitting corrosion in Cl^−^ bearing media [54].

Figure 10a and Figure 11a show the initial sputtering stage. Cr2p_3/2_ is primarily made up of Cr(OH)_3_, CrO_4_^2−^, and Cr_2_O_3_, and Cr(OH)_3_ and CrO_4_^2−^ gradually decreased to 30 s and then vanished as the sputtering progressed; Cr_2_O_3_ gradually increased and then decreased throughout the sputtering; and Crmet gradually increased. Figure 10b and Figure 11b reveal that at the preliminary stage of sputtering, Fe2p_3/2_ is dominantly Fe_2_O_3_, Fe_3_O_4_, FeOOH, and FeO, and as sputtering advances, FeOOH and FeO gradually diminish until they all vanish after 30 s. Fe_2_O_3_ and Fe_3_O_4_ gradually increase and then diminish during 45 s. This indicates that the outside FeO and FeOOH are converted from the inside Fe_3_O_4_ with Fe_2_O_3_, and Fe_3_O_4_ is a hybrid oxide made up of Fe^2+^ and Fe^3+^, which can be expressed as FeO + Fe_2_O_3_ [55]. Figure 10c and Figure 11c show the molecular make-up of Mo3d. The MoO_4_^2−^ concentration is at its highest at the start of sputtering and decreases first as the duration of the sputtering increases. A modest increase is followed by a decrease in MoO_2_, showing that MoO_4_^2−^ may be converted from MoO_2_.

With a growing sputtering period, the values of Cr_2_O_3_ to Cr(OH)_3_ + CrO_4_^2−^ (Figure 11a), Fe_OX_^3+^ (Fe^3+^ oxide) to Fe_OX_^2+^ (Fe^2+^ oxide) (Figure 11b), and Mo ions/Mo (Figure 11c) in the passive film on the sample surface continuously improved. In other words, the internal layer of the passive film primarily consists of Cr_2_O_3_, Fe_OX_^3+^ and MoO_2_, and the external layer of the passive film is made up of primarily CrO_4_^2−^, Cr(OH)_3_, Fe_OX_^2+^, MoO_2_, and MoO_4_^2^. It is hypothesized that during the passivation process, Cr, Fe, and Mo ions are liberated from the steel surface and that because Cr has a lower Gibbs free energy than other elements, it is more easily able to form crystals and grow [56]. Meanwhile, Cr(OH)_3_ has a significantly smaller standard free energy [57], so it forms first on the surface of the passive film (reaction (7)), and Cr(OH)_3_ subsequently occurs in reactions 8 and 9, producing CrO_4_^2−^ and Cr_2_O_3_.

4 Cr^3+^ + 3 O_2_ + 6 H_2_O + 12 e^−^ → 4 Cr(OH)_3_ (7)

Cr(OH)_3_ + 5 OH^−^ → CrO_4_^2−^ + 4 H_2_O + 3 e^−^ (8)

4 Cr(OH)_3_ + 4 Cr + 3 O_2_ → 4 Cr_2_O_3_ +6H_2_O (9)

The values of Cr_2_O_3_ to Cr(OH)_3_ (Figure 10a), Fe_OX_^3+^ to Fe_OX_^2+^ (Figure 11b), and Mo ions/Mo (Figure 11c) exhibited an increasing trend as the Mo content increased and the Cr content decreased. From Figure 10c and Figure 11c, a trend of increasing CrO_4_^2−^ and MoO_4_^2−^ content with increasing Mo content is observed. This may be because the bipolar charge passivation model applies to this system [5], where MoO_4_^2−^ transforms the passive film into a bipolar film with an internal negative ion-selective layer and an outer positive ion-selective layer. This repels Cl^−^ from entering the passive film and causes deprotonation (reaction (10)) in the internal layer of the bipolar film junction. This in turn causes protons to pass through the positive ion-selective outer layer toward the solution. When OH^−^ is deprotonated to form O^2−^ ions, it speeds up the development of more and denser Cr_2_O_3_ [10]. Additionally, MoO_2_ can help maintain the homogeneity of the passive film. With a denser passive film, an improved protective effect on the surface is achieved [58]. This explicates the reason why the R and n values of the passive film improve as the Mo concentration increases and the Cr concentration drops, while the N_D_ and N_A_ of the passive film drop.

2Cr(OH)_3_→Cr_2_O_3_ + 2O^2−^ + 4 H^+^ (10)

### 3.6. Influence of Cr and Mo Doping on Passive Film Performance against NaCl Attraction

**Figure 13:**
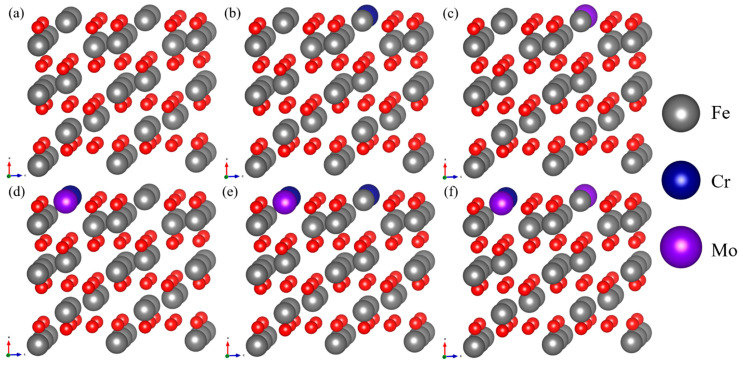
Models constructed: (**a**) Fe_2_O_3_, (**b**) 1Cr, (**c**) 1Mo, (**d**) 1Cr1Mo, (**e**) 2Cr1Mo, and (**f**) 1Cr2Mo.

Six models were built using Fe_2_O_3_ to examine the synergistic interaction of Cr and Mo in passive films. These models include Fe_2_O_3_, 1Cr, 1Mo, 1Cr1Mo, 2Cr1Mo, and 1Cr2Mo. To simulate the synergistic interaction, the Fe atoms on the surface of these models are replaced with Cr or Mo atoms. There is just one structure per model since the four Fe atoms are in equivalent positions on the surface. Figure 13 shows the six models constructed.

The adsorption energy (E_ads_) can be calculated from the energy E_NaCl+Film_ of the (0001) surface after adsorption of 1 NaCl, the energy E_Film_ of the (0001) surface without NaCl adsorption, and the energy E_NaCl_ of a single NaCl according to Equation (11). The charge transfer (ΔQ) resulting from adsorption can be calculated from the charge Q_ads_ of Cl in the adsorbed NaCl molecule, and the charge Q_NaCl_ of Cl in the NaCl molecule alone, with a value of 7.84794 eV. A positive result represents losing electrons and a negative result represents gaining electrons, according to Equation (12). The work function (W) can be calculated from the vacuum energy level E_vacuum_. The Fermi energy E_F_ based on equation 13 can be used to compute the work function (W). The bigger the W, the less susceptible to corrosion the material [59].

E_ads_ = E_NaCl+Film_ − E_NaCl_ − E_Film_ (11)

ΔQ = Q_NaCl_ − Q_ads_ (12)

W = E_vacuum_ − E_F_ (13)

Upon completion of the adsorption calculation, the conditions around the surface atoms was altered, leading to a relaxation phenomenon on the surface where the atoms were shifted along various orientations. Our study discovered that surface displacements when NaCl was adsorbed mainly manifested in the a-orientation, with only minor shifts in other orientations. Therefore, we mark the initial location of the adsorbed atoms on the surface in the a-orientation as d_1_, which, after computation, changes its coordinates to d_2_. To measure the degree of shift in the a-orientation, we use Δd = d_2_ − d_1_. When Δd > 0, this represents a shift of the atoms in the a-orientation, which is far from the surface orientation, and when Δd < 0, this represents a shift in the atoms toward the bulk phase.

The E_ads_, adsorption distance, bond distance, W, and Δd values for the respective adsorption positions for the respective models are listed in Table 6, where negative values for E_ads_ can be found, suggesting that the process is exothermic. In contrast, 1Cr1Mo has a W value of pure Fe_2_O_3_ enhancement of 0.19484 eV, which is larger than the combined enhancement of 1Cr and 1Mo. In contrast, 1Cr and 1Mo each have a minor enhancement in the W value of pure Fe_2_O_3_ of 0.03787 and 0.05747 eV, respectively, and 2Cr1Mo improves the W value of pure Fe_2_O_3_ by 0.27267 eV, which is more than the combination of the 1Cr1Mo and 1Mo lifts. Additionally, 1Cr2Mo increases the W value of pure Fe_2_O_3_ by 0.35261 eV, which is significantly greater than the sum of the lifts from 1Cr1Mo and 1Mo. This, along with the synergistic interaction between Cr and Mo, shows that the passive film’s corrosion resistance increases as the concentration of Cr and Mo increases, with Mo’s influence being more evident.

According to Table 6, it is observed that Fe_2_O_3_, 1Cr, and 1Mo have various adsorption energies at various adsorption sites. When Cr replaces Fe, the adsorption energy of the Cr atom increases by 27.45964 kJ·mol^−1^, but for the adjacent Fe atom, the change in adsorption energy is smaller, merely improving from −82.79196 kJ·mol^−1^ to −80.85261 kJ·mol^−1^. Despite the small change in the adsorption energy of Mo, with an increase of only 9.59351 kJ·mol^−1^, its influence on the adsorption energy of nearby Fe atoms is greater, leading to an increase of 19.94442 kJ·mol^−1^. This shows that although Mo improves the corrosion resistance of the passive film mostly by enhancing the corrosion resistance of nearby atoms’ oxide, Cr enhances the corrosion resistance of the passive film through its oxide.

It is evident from Table 6 that the adsorption energy is more significantly improved due to the presence of both Cr and Mo in 1Cr1Mo, 2Cr1Mo, and 1Cr2Mo. In the 1Cr1Mo model, the adsorption energy at Cr improves from −55.33232 kJ·mol^−1^ in the 1Cr model to −41.02553 kJ·mol^−1^ and the adsorption energy at Mo improves from −73.19845 kJ·mol^−1^ in the 1Mo model to −65.37545 kJ·mol^−1^, while the adsorption energy at Fe in 1Cr1Mo increases from −80.85261 kJ·mol^−1^ and −62.84754 kJ·mol^−1^ in the 1Cr and 1Mo models to −56.41266 kJ·mol^−1^. The improved adsorption energies at all three adsorption locations, particularly in the 1Cr2Mo model, are a strong indication that Mo plays a more significant role than in the 2Cr1Mo model and that the improvement is the result of a synergistic process rather than merely replacing atoms.

Based on the adsorption data from the six models, it is evident that for the identical atoms, the bond distance is positively proportional to the adsorption energy, while Δd and ΔQ are inversely proportional to the adsorption energy. The greater the bond distance, the weaker the ΔQ and Δd, suggesting weaker interatomic interactions and demonstrating that the stability of the passive film structure is less disrupted by NaCl [60]. When Mo is used as the adsorption site, ΔQ is marginally higher compared with Fe as the adsorption point, probably due to the larger quantity of electrons external to the nucleus of Mo.

## 4. Conclusions

In a 3.5% NaCl solution at 50 °C with super austenitic stainless steel, the passive film formed is found to be double-layered. The internal layer is primarily composed of Cr_2_O_3_ and Fe_OX_^3+^, and the external layer is primarily composed of CrO_4_^2−^, Cr(OH)_3_, Fe_OX_^2+^, and MoO_2_ with MoO_4_^2−^. The concentration of Cr_2_O_3_ and Fe_OX_^3+^ rises to the same thickness as the Mo concentration in the steel increases from 4.02 wt.% to 6.02 wt.% and the Cr concentration decreases from 27.96 wt.% to 21.45 wt.%, resulting in a thicker, more uniform, and a denser passive film. This results in a lower N_A_ and N_D_ and more R and E_FB-n_ in the passive film, decreasing the space charge conductivity and thus lowering the passive film’s ability to interact with external Cl^−^. There are fewer pitting pits as a result of a more uniform, higher Cr_2_O_3_ concentration passive layer that forms with fewer active regions where pitting corrosion originates. Fewer active areas in the passive film slow down the emergence and growth of pitting, and MoO_4_^2−^ also slows down corrosion, resulting in a decrease in the maximum pitting depth and area. In terms of adsorption energy, although Mo does this primarily by increasing the oxide adsorption energy of nearby atoms, Cr can increase the corrosion resistance of the passive film through its oxide. The increase in W and adsorption energy may not act independently by merely replacing atoms but rather in a complex synergy of interactions, as evidenced by the fact that the increase in adsorption energy when Cr and Mo are doped together is significantly greater than the summation effect when Cr and Mo are doped separately. Therefore, increasing the Mo concentration to reduce the Cr concentration can enhance the corrosion resistance of stainless steel and improve its service life in marine applications.

## Figures and Tables

**Table 1:** Chemical constituent of samples/wt.%.

NO.	C	Si	Mn	Ni	Cr	Mo	N	Fe	PREN
Cr2Mo1	0.0067	0.35	0.41	24.37	27.96	4.02	0.29	Bal.	45.87
Cr1Mo1	0.0063	0.33	0.39	24.32	24.69	5.01	0.30	Bal.	46.02
Cr1Mo2	0.0065	0.31	0.42	24.45	21.45	6.02	0.29	Bal.	45.96

**Table 2:** Parameters of the fitted impedance of EIS in a 3.5% NaCl medium at 50 °C.

NO.	R_s_/Ω·cm^2^	Q/×10^−6^ Ω^−1^·cm^−2^·s^n^	n	R/10^6^ Ω·cm^2^	C/×10^−6^ F·cm^−2^	χ^2^/×10^−4^
Cr2Mo1	2.586	3.749	0.9418	6.138	4.551	8.497
Cr1Mo1	2.647	3.297	0.9498	7.702	3.912	6.552
Cr1Mo2	2.667	2.764	0.9579	9.982	3.198	1.618

**Table 3:** N_A_, N_D_, and E_FB_ in 3.5% NaCl liquor at 50°C.

NO.	Acceptor Density, N_A_ × 10^−20^/cm^−3^	E_FB-p_ /V_SCE_	Donor Density, N_D_ × 10^−20^/cm^−3^	E_FB-n_ /V_SCE_
Cr2Mo1	5.238906	0.41294	2.090764	−0.09251
Cr1Mo1	3.941489	0.21721	1.715653	−0.03904
Cr1Mo2	3.313983	0.13984	1.419965	0.00957

**Table 4:** Passive film thickness after immersion in 3.5% NaCl medium at 50 °C for 24 h.

NO.	Position 1 /nm	Position 2 /nm	Position 3 /nm	Position 4 /nm	Position 5 /nm	Average Value /nm
Cr1Mo2	6.33	6.15	6.51	6.69	6.35	6.41
Cr2Mo1	8.69	8.98	9.67	9.86	9.45	9.33
Cr1Mo1	10.21	10.85	10.82	10.52	9.52	10.38
Cr1Mo2	11.51	14.81	13.73	12.94	11.12	12.82

**Table 5:** Compound binding energy of every element.

Element	Valence state	Binding energy/eV
Cr	Cr_met_	574.0 ± 0.1
	CrO_4_^2−^	578.3 ± 0.1
	Cr(OH)_3_	577.3 ± 0.1
	Cr_2_O_3_	576.4 ± 0.1
Fe	Fe_met_	707.1 ± 0.1
	FeOOH	711.7 ± 0.1
	Fe_2_O_3_	710.4 ± 0.1
	FeO	709.5 ± 0.1
	Fe_3_O_4_	708.2 ± 0.1
Mo	(Mo_met_): Mo3d_5/2_, Mo3d_3/2_	228.0 ± 0.1, 230.9 ± 0.1
	MoO_2_: Mo3d_5/2_, Mo3d_3/2_	231.0 ± 0.1, 234.2 ± 0.1
	MoO_4_^2−^: Mo3d_5/2_, Mo3d_3/2_	232.3 ± 0.1, 235.7 ± 0.1

**Table 6:** Correlation data after adsorption of different models.

Models	Adsorption Site	E_ads_/kJ mol^−1^	Bond Distance/Å	W/eV	Δd/Å	ΔQ/e
Fe_2_O_3_	Fe	−82.79196	2.28692	4.19675	0.01537	0.16721
1Cr	Cr	−55.33232	2.31747	4.23462	0.01329	0.14364
	Fe	−80.85261	2.28793		0.01511	0.16583
1Mo	Mo	−73.19845	2.40037	4.25422	0.00749	0.17546
	Fe	−62.84754	2.30214		0.01394	0.15267
1Cr1Mo	Cr	−41.02553	2.37406	4.39159	0.01214	0.11063
	Mo	−65.37545	2.40702		0.00747	0.1747
	Fe	−56.41266	2.31104		0.01355	0.14614
2Cr1Mo	Cr	−39.24731	2.38359	4.46942	0.01209	0.10589
	Mo	−57.56595	2.40955		0.00737	0.17381
	Fe	−51.24136	2.32304		0.01281	0.14177
1Cr2Mo	Cr	−35.0946	2.39966	4.54936	0.01152	0.09327
	Mo	−46.09967	2.41147		0.00674	0.17169
	Fe	−43.21863	2.36328		0.01229	0.12877

## Data Availability

Data are contained within the article.
